# The Efficacy and Safety of Brain Radiotherapy Combined With Immune Checkpoint Inhibitors (ICIs) for Small‐Cell Lung Cancer (SCLC) Patients With Brain Metastases (BMs)

**DOI:** 10.1111/1759-7714.70112

**Published:** 2025-06-25

**Authors:** Jie Xu, Yanling Yang, Tingting Chen, Dongmin Liu, Yajing Yuan, Liming Xu

**Affiliations:** ^1^ Department of Lung Cancer Tianjin Lung Cancer Center, Tianjin Medical University Cancer Institute and Hospital, National Clinical Research Center for Cancer, Tianjin's Clinical Research Center for Cancer, Key Laboratory of Cancer Prevention and Therapy Tianjin China; ^2^ Department of Radiation Oncology Tianjin Medical University Cancer Institute and Hospital, National Clinical Research Center for Cancer, Key Laboratory of Cancer Prevention and Therapy, Tianjin, Tianjin's Clinical Research Center for Cancer Tianjin China; ^3^ Department of Anesthesia Tianjin Medical University Cancer Institute and Hospital, National Clinical Research Center for Cancer, Key Laboratory of Cancer Prevention and Therapy, Tianjin, Tianjin's Clinical Research Center for Cancer Tianjin China; ^4^ Department of Radiation Oncology Tianjin Cancer Hospital Airport Hospital Tianjin China

**Keywords:** brain metastases, carcinoma, efficacy, radiotherapy, safety, small‐cell lung cancer

## Abstract

**Objective:**

This study was designed to evaluate the efficacy and safety of brain radiotherapy combined with immune checkpoint inhibitors (ICIs) retrospectively in small‐cell lung cancer (SCLC) patients with brain metastases (BMs).

**Methods:**

A retrospective analysis was conducted on 42 SCLC patients with BMs, who received brain radiotherapy combined with ICIs at our Hospital from 2020 to 2024. They received chemotherapy plus ICIs regimens and brain radiotherapy, and received concurrent/sequential thoracic radiotherapy. This study investigated the forms of WBRT vs. WBRT+ simultaneous integrated boost (SIB, different doses of radiation being delivered simultaneously to different parts of the tumor) combined with ICIs on overall survival (OS), intracranial local control (iLC), and radiotherapy adverse reactions (side effection). The Kaplan–Meier method was used for survival rate analysis. The log‐rank test was used to compare the survival curves between different groups, and the chi‐square (χ^2^) test was used to compare categorical data.

**Results:**

In all the patients, the median follow‐up time was 19.2 (range: 9.79–36.8) months. The 2‐year OS rate and iLC rate were 42.3% and 68.8%, respectively. A total of 26 patients died of disease progression; 2 patients developed radiation‐induced brain necrosis. The results showed that there was no significant difference in radiation‐induced brain necrosis between the two groups. The WBRT patients suffered high rates of headache, dizziness, nausea, and radiodermatitis. The 2‐year OS and iLC were brilliant.

**Conclusions:**

When brain radiotherapy combined with ICIs, even WBRT or WBRT + SIB had well OS and iLC with tolerable side reactions. Its indications needed to be considered from multiple perspectives. Further evaluation of brain radiotherapy combined with ICIs in SCLC BMs is required. Further prospective studies should be conducted to verify the conclusions.

## Graphical abstract

The median overall survival rate (OS) and intracranial local control (iLC) was 11.3 months (95% confidence interval: 7.0–12.6 months) and 14.3 months (95% confidence interval: 12.7–15.9 months). The 2‐year OS and iLC rate was 42.3% and 68.8%, respectively.

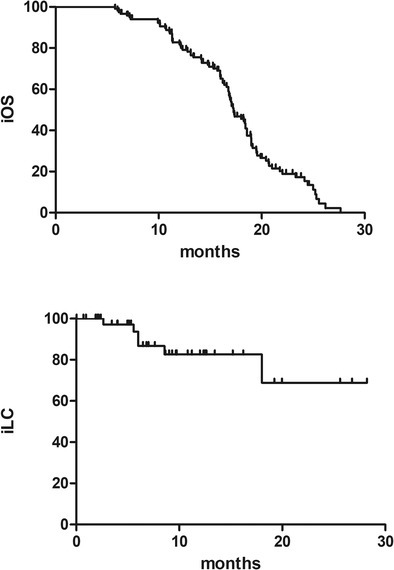

## 1 Introduction

Small‐cell lung cancer (SCLC) is a neuroendocrine lung cancer, and it accounted for 15%–20% of lung cancers. It is mainly characterized by invasive growth with early metastases. Approximately 80% of patients have distant metastases at the time of diagnosis [1]. It was highly malignant with poor prognosis, and the 5‐year survival rate was less than 7% [2]. BMs are the most common type of distant metastasis. The treatment methods for SCLC with BMs include surgery, chemotherapy, radiotherapy, and the emerging immunotherapy. For a long time, whole brain radiation therapy (WBRT) has been regarded as one of the standard treatment options for ES‐SCLC brain metastasis (BM), but some recent studies have shown that WBRT does not prolong the patient's final OS [3].

In the past period, relying on traditional chemoradiotherapy treatment regimens, the median survival was only 4–6 months [4]. In recent years, studies have shown that immunotherapy targeting programmed cell death ligand‐1 (PD‐L1) combined with platinum‐based chemotherapy has shown an superior outcome of the overall survival (OS) in patients with extensive‐stage SCLC (ES‐SCLC) [5]. However, the presence of the blood–brain barrier Some systemic drugs cannot reach intracranial blood levels, which makes the central nervous system a refuge for metastasis [6]. Studies have found functional lymphatic vessels in the dura mater sinus [7], proving that immunotherapy has an inestimable effect on SCLC‐BM. Radiotherapy reprograms the tumor immune microenvironment through a variety of mechanisms and works synergistically with immunotherapy [8]. Therefore, the integration of radiotherapy and immunotherapy as a treatment method has broad potential, and it is possible to improve the survival results of patients diagnosed with SCLC‐BMs. However, the specific role of RT‐immune checkpoint inhibitors (ICIs) in SCLC‐BMs and whether the efficacy and safety of the RT‐ICIs program in SCLC‐BMs are better than the original use of traditional RT alone are still unclear. Therefore, ICIs and brain radiotherapy require further research. This study analyzes the efficacy and safety of brain radiotherapy combined with ICIs retrospectively, in order to optimize the suitable brain radiotherapy strategy for SCLC patients with BMs.

## 2 Materials and Methods

### 2.1 Inclusion Criteria

A retrospective analysis was conducted on 42 SCLC patients with BMs, who received brain radiotherapy at the Tianjin Medical University Cancer Institute and Hospital from 2020 to 2024. The clinical data of all patients were obtained from outpatient or inpatient records. Patients with a radiological diagnosis of BM were eligible for inclusion in this study. This study was approved by the Ethics Committee of the Tianjin Medical University Cancer Institute and Hospital (approval number: bc202244), and patients were exempted from signing the informed consent form. Patients underwent standardized physical examinations, computed tomography (CT) of the neck, chest, and abdomen, enhanced magnetic resonance imaging (MRI) of the brain, and either emission computed tomography (ECT) or positron emission tomography/computed tomography (PET/CT). All SCLC patients underwent histopathological evaluation, and the diagnosis of SCLC conformed to the pathological diagnostic criteria for SCLC. ES‐SCLC in this study referred to those classified as ES‐SCLC according to the Veterans Administration Lung Cancer Staging System and the 8th Edition Lung Cancer. Stage Classification.

### 2.2 Treatment Strategy

All patients received chemotherapy plus ICIs regimens and brain radiotherapy, and received concurrent/sequential thoracic radiotherapy. The majority of patients were treated with sequential immunotherapy in the study. **Chemotherapy regimens**: The chemotherapy regimens were platinum‐based first‐line doublet chemotherapy regimens (cisplatin or carboplatin+etoposide). **ICIs regimens**: such as durvalumab, atezolizumab, adebrelimab, or serplulimab as the first‐line or second‐line ICIs regimens. **Thoracic radiotherapy**: Intensity‐modulated radiotherapy (IMRT) techniques were adopted. The gross tumor volume (GTV) included postchemotherapy pulmonary lesions and mediastinal enlarged lymph nodes. The clinical target volume (CTV) was expanded by 0.5 cm outward on the basis of the GTV, including the entire lymphatic drainage area where the lymph nodes with metastasis confirmed by prechemotherapy imaging were located. The planning target volume (PTV) was uniformly expanded by 0.5–1.0 cm in all directions on the basis of the CTV. **Brain radiotherapy**: The CTV of whole brain encompassed the whole‐brain parenchyma. The PTV of whole brain was defined as the 3–5 mm margin to the CTV. The GTV encompassed contrast‐enhancing tumor on MRI and was reviewed by the radiation oncologist or the neurosurgeon based on the tumor volume, tumor location, and neurological symptoms. The PTV of brain metastases (PTVbm) was defined as the 1–3 mm margin to the GTV. The prescribed dose was 35–50 Gy in 10 fractions with BMs, and 30 Gy in 10 fractions with whole brain. The 42 patients were divided into the WBRT group (19 cases) and WBRT+SIB group (23 cases) according to the radiotherapy methods for BMs. Twenty‐three patients received SIB through IMRT simultaneously (35–50 Gy in 10 fractions, 3.5–5 Gy per fraction) with WBRT. The SIB dose was selected according to the size of the tumor: the prescribed dose for metastatic tumors with the longest diameter (*r*) ≤ 10 mm was 50 Gy [biological effective dose (BED) = 75Gy]; for those with 10 mm < *r* ≤ 30 mm, the prescribed dose was 40 Gy (BED = 56 Gy); for those with 30 mm < *r* ≤ 40 mm, the prescribed dose was 35 Gy (BED = 47.25 Gy).

### 2.3 Evaluation of Efficacy and Follow‐Up

Acute adverse reactions were graded according to the (Common Terminology Criteria for Adverse Events [CTCAE] version 5.0), and the late adverse reactions were graded based on the criteria of the Radiation Therapy Oncology Group (RTOG). The efficacy of solid tumors was evaluated according to the Response Evaluation Criteria in Solid Tumors 1.1(RECIST 1.1). The baseline assessment was repeated every two cycles and then repeated every 6–8 weeks after the end of treatment until disease progression. OS was defined as the time from the start of treatment until the occurrence of an event or the last follow‐up. Intracranial local control (iLC) was defined as the time from the date when BM was confirmed by imaging examinations (enhanced MRI or enhanced CT) to the time of the occurrence of an event or death due to any reason or the last follow‐up time.

### 2.4 Statistical Analysis

Statistical analysis was performed using SPSS 26.0 software. Patient characteristics were summarized using descriptive statistics. Categorical data were recorded as the number of cases (%), and continuous data were recorded as the median (range). The Kaplan–Meier method was used for survival rate analysis. The log‐rank test was used to compare the survival curves between different groups, and the chi‐square (χ^2^) test was used to compare categorical data. Univariate survival analysis was performed using a Cox proportional hazards model to determine associations between OS and the important clinical factors. The difference in toxicity rate is calculated by Fisher's precise test. In this study, all P‐values were from one‐sided tests, and a difference was considered statistically significant when *p* < 0.05.

## 3 Result

### 3.1 Clinical Features

The patient characteristics of 42 patients were shown in Table 1. The median age of the 42 enrolled patients was 56.5 years old (range 39–74). Thirty‐five patients (83.3%) were male. Twenty‐eight patients (66.7%) had heavy smoking (smoking index ≥ 400). Thirty‐six patients (85.7%) had a Karnofsky performance status (KPS) ≥ 80. Forty‐one patients (97.6%) received more than four cycles of chemotherapy and 38 patients (90.5%) responded to chemotherapy. All patients received ICIs treatment with brain radiotherapy.

**TABLE 1:** Distribution of the 42 Patients' Treatment and Clinical Characteristics.

Characteristic	Number (Ratio, %)	Characteristic	Number (Ratio, %)
Age(years)		Number of BMs	
< 65 years	5 (11.9)	1	13 (31.0)
≥ 65 years	37 (88.1)	2–3	11 (26.2)
Gender		> 3	18 (42.8)
Male	35 (83.3)	Maximum diameter of the largest tumor(cm)	
Female	7 (16.7)	≤ 2.0	25 (59.5)
Smoke index		> 2.0	17 (40.5)
≥ 400	28 (66.7)	Interval from diagnosis of SCLC to BMs (mths)	
< 400	14 (33.3)	≤ 10	17 (40.5)
Family history of tumors		> 10	25 (59.5)
No	36 (85.7)	Extracranial disease control status	
Yes	6 (14.3)	Yes	31 (73.8)
Weight loss		No	11 (26.2)
> 5%	12 (28.6)	GPA	
≤ 5%	30 (71.4)	< 2	13 (31.0)
KPS		≥ 2	29 (69.08)
≥ 80	36 (85.7)	Immunotherapy(ICI)	
< 80	6 (14.3)	Yes	42 (100)
TRT dose		No	0 (0)
< 50Gy	0 (0)	Targeted therapy (anti‐angiogenic therapy)	
≥ 50Gy	42 (100)	Yes	5 (11.9)
Stage		No	37 (88.1)
LS‐SCLC	4 (90.5)	Brain radiotherapy	
ES‐SCLC	38 (9.5)	WBRT	19 (45.2)
		WBRT+SIB	23 (54.8)

### 3.2 Survival and Adverse Reactions

**FIGURE 1:**
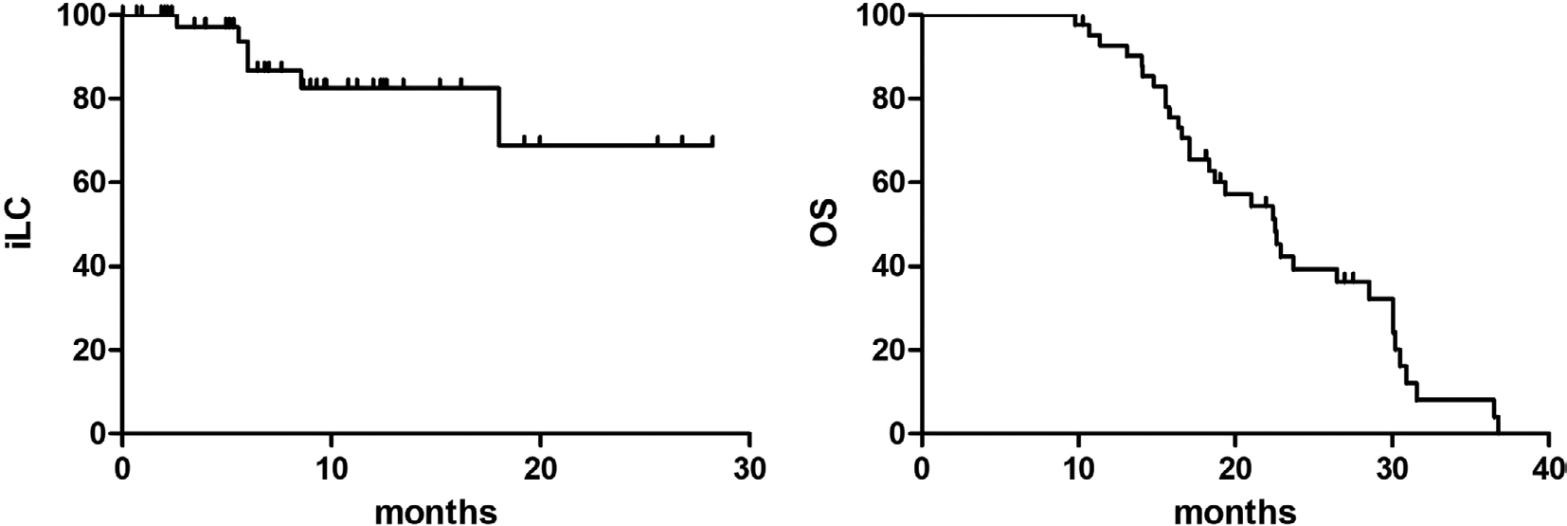
The OS and iLC in 42 SCLC with BMs patients.

The median follow‐up time was 19.2 (range: 9.79–36.8) months. The median OS rate and iLC was 11.3 months (95% confidence interval: 7.0–12.6 months) and 14.3 months (95% confidence interval: 12.7–15.9 months). The 2‐year OS and iLC rate was 42.3% and 68.8%, respectively (Figure 1). A total of 26 patients died of disease progression, two patients developed radiation‐induced brain necrosis. The predictive significance of patients' clinical and disease characteristics for OS. In the univariate analysis of the influencing factors of OS, KPS≥ 80 (*p* = 0.002), the local stage (LS) (*p* = 0.014), and WBRT+SIB (*p* = 0.014) were significantly correlated with an increase in the survival rate (Table 2).

**TABLE 2:** Clinical and Treatment Characteristics and Survival‐Related Factors on OS in Univariate and Multivariate Analysis.

Characteristic	Univariate analysis	
	HR(95% CI)	*p*
Age(≥ 65 years. v.s. < 65 years)	0.100 (0.006–1.724)	0.113
Gender(female v.s. male)	3.847 (0.708–20.905)	0.119
Smoke index (≥ 400v.s. < 400)	0.496 (0.167–1.473)	0.206
Family history of tumors (no v.s.yes)	3.593 (0.786–16.433)	0.099
Weight loss (≤ 5%v.s. > 5%)	1.780 (0.557–5.692)	0.331
KPS(≥ 80v.s. < 80)	12.856 (2.461–67.158)	0.002
Stage(LS v.s. ES)	16.717 (1.766–158.225)	0.014
Number of BMs (1v.s.2‐3v.s. > 3)	1.527 (0.763–3.053)	0.232
Maximum diameter of the largest tumor (cm)(≤ 2.0v.s. > 2.0)	3.019 (0.984–9.259)	0.053
Interval from diagnosis of SCLC to BMs (mths)(≤ 9v.s > 9)	0.356 (0.113–1.117)	0.077
Extracranial disease control status (yes v.s. no)	1.549 (0.353–6.794)	0.562
GPA(< 2 v.s. ≥ 2)	1.270 (0.131–12.328)	0.837
BM Radiotherapy (WBRTv.s.WBRT+SIB)	0.196 (0.0530.718)	0.014

The diagnosis of radiation‐induced brain necrosis was made by the radiotherapy doctors and radiologists based on enhanced cranial MRI combined with magnetic resonance spectroscopy. The majority of patients experienced treatment‐related adverse reactions, mainly including nausea, vomiting, dizziness, headache, leukopenia, radiation‐induced brain necrosis, etc. (Table 3). One patient suffered from radiation‐induced brain necrosis because of an excessively high SIB dose. While 1 patient with more than 10 BMs suffered from that side effect. The WBRT patients suffered high rates of dizziness (*p* = 0.016), nausea (*p* = 0.001), and radiodermatitis (*p* = 0.046) because WBRT patients had more BMs and worse symptoms.

**TABLE 3:** The Adverse Reaction Incidence of Brain Radiotherapy Combined With ICIs in SCLC With SMs.

Side effects	WBRT + SIB(23 cases)		WBRT(19 cases)		*p*
	Grade 1or2	Grade 3	Grade 1or 2	Grade 3	
Fatigue	3	0	4	0	0.745
Headache	20	3	19	0	0.102
Dizziness	17	6	19	0	0.016
Nausea	23	0	12	7	0.001
Vomiting	4	0	4	0	
Fever	1	0	1	0	
Leukopenia	12	3	10	2	0.825
Thrombocytopenia	2	0	2	0	
Radiodermatitis	2	0	0	2	0.046
Disturbance of consciousness	0	0	0	0	
Radiation‐induced brain necrosis	0	1	1	0	0.157

## 4 Discussion

With the promotion of comprehensive treatment for SCLC and the application of ICIs, the OS of SCLC patients has significantly increased, reaching more than 12 months. Therefore, the treatment approach forBMs should also be adjusted accordingly, requiring more aggressive treatment. Previous research by the team from Tianjin Medical University Cancer Institute and Hospital has confirmed that local dose‐escalated radiotherapy for BMs can extend the survival of SCLC patients with BMs [9] Based on this, this study further investigated the forms of WBRT v.s. WBRT+SIB combine with ICIs on OS, iLC, and radiotherapy adverse reactions.

The results showed that there was no significant difference in radiation‐induced brain necrosis between the two groups (Table 3). The WBRT patients suffered high rates of headache, dizziness, nausea, and radiodermatitis because of WBRT patients with more BMs and worse symptoms. The 2‐year OS and iLC were brilliant.

More than 50% of SCLC patients may develop BMs [10], and the prognosis of such patients is poor. The treatment of BMs in SCLC patients is different from that in other solid tumors due to its diffusivity and invasiveness [11]. Even in stage I–II SCLC patients who have undergone surgical resection, the cumulative incidence of BMs is as high as 30% [12]. The metastasis rate of BM in SCLC is so high; in order to improve the survival of such patients, we will further improve the treatment options for such patients.

The treatment plan for BMs was adopted according to the time of BMs occurrence, initial diagnosis (synchronous or metachronous BMs), and patient symptoms. In addition, the radiotherapy plan also takes into account the patient's extracranial disease status at that time and whether they have received radiotherapy in the past. WBRT improved the time to progression (TTP) (hazard ratio, HR = 0.38, *p* < 0.001), but did not significantly improve OS (median PSM: 6.5 months [Stereotactic radiosurgery, SRS] vs. 5.2 months [WBRT], *p* = 0.003) [13]. Several clinical trials have reported the role of dose‐escalated radiotherapy with WBRT in the treatment of BMs [13] Andrews et al. recruited 331 SCLC patients with 1–3 BMs. Univariate analysis showed that patients with solitary BMs had a survival advantage in the WBRT and stereotactic radiotherapy groups (median survival time of 6.5 vs. 4.9 months, *p* = 0.0393). During the 6‐month follow‐up, patients in the stereotactic surgery group were more likely to achieve stable or improved KPS scores compared to those allocated to WBRT alone (43% vs. 27%; *p* = 0.03) [14]. it also improved the survival of patients with a single BM (median OS 6.5 vs. 4.9 months) (*p* = 0.039) [15].

SIB can shorten the treatment time and increase the dose to the BM lesions. In addition, some studies have shown that synchronous dose‐escalated radiotherapy for BM lesions can effectively control intracranial lesions and improve survival [16, 17]. Recently, Qing et al. [18] reported that the median OS of BM patients treated with WBRT+SIB was 10 months. SCLC patients have a high risk of intracranial progression, and SIB may be more suitable. Compared with WBRT alone in this study, WBRT+SRS significantly improved the local control rate of intracranial metastases at 1 year (82% vs. 71%, *p* = 0.013); the conclusions are similar. Therefore, from the above and the conclusions of this study, it can be seen that in SCLC‐MBs, WBRT+SIB has a better curative effect than simple WBRT.

In recent years, phase I/II trials of concurrent immunotherapy and prophylactic cranial irradiation (PCI) (pembrolizumab administered within 21 days of PCI) in limited‐stage SCLC have found that compared with patients who did not receive PCI, the median overall survival (mOS) of patients who received concurrent ICIs and PCI was significantly prolonged (39.5 vs. 30.0 months, *p* < 0.05). However, most clinical trials of nonconcurrent PCI combined with immunotherapy have yielded negative results [19]. SRS combined with ICIs can improve local control and survival outcomes in nonsmall‐cell lung cancer (NSCLC) BMs, but there is still insufficient evidence on the optimal dosage and sequence (whether synchronous or sequential) of radiotherapy in combination with ICIs. We need to study further. Schapira et al. found that a retrospective study evaluating SRS in combination with ICIs (synchronous versus sequential ICIs treatment) had superior OS [20].

Regarding the toxicity of combined treatment, some studies have shown that the combined treatment for NSCLC BMs were well‐tolerated. Most Researches indicated that the most common neurological adverse reactions of radiotherapy combined with ICIs for NSCLC BMs are fever, nausea, seizures, and fatigue [21]. The adverse reactions of the combined treatment are mainly grade 1–2, rarely grade 3–4, and there is no adverse reactions grade 5. The application of this treatment regimen in SCLC patients with BMs has not been clearly defined. Currently, most of the conclusions for SCLC patients with BMs relyed on subgroup analyses of classic trials such as Impower133, CASPIAN, and KEYNOTE—604. The addition of ICIs did not lead to new aor potential dverse reactions in SCLC patients with BMs. A retrospective study also showed that compared with PCI/WBRT, PCI/WBRT + atezolizumab did not increase the risk of neurotoxicity [22]. This study indicated that brain radiotherapy combined with ICIs for SCLC BMs was effective and well‐tolerated. In the Impower133 study, patients were allowed to undergo PCI during the maintenance phase after the completion of concurrent chemoimmunotherapy, and the proportion of patients receiving PCI in each study group was 11% [23]. In the KEYNOTE‐604 study, patients who showed certain efficacy after four cycles of treatment, at the discretion of the researchers, receive PCI (25 Gy/10 fractions). Eventually, 27 patients (11.8%) in the pembrolizumab+EP group received PCI [24]. Neither the Impower133 nor the KEYNOTE‐604 trial found safety in patients receiving PCI combined with ICIs. Currently, there are no large‐scale prospective clinical studies, and this clinical regimen remains to be further explored. Therefore, more research is preferred.

In our study, most patients experienced related adverse reactions, including nausea, vomiting, dizziness, headache, leukopenia and other neurotoxicities. the safety characteristics are generally consistent with the previously reported known safety data of brain radiation therapy, chemotherapy and immunotherapy, or combination use [21, 22, 23, 24]. The incidence of adverse events is acceptable and in line with our expectations. It is also worth noting that most of the patients in this study had nervous system symptoms at baseline, so it is difficult to determine whether acute nervous system symptoms are due to research treatment or BM.

## 5 Conclusions

Radiotherapy, as a fundamental treatment method, has been used for ES‐CLC for many years. When brain radiotherapy is combined with ICIs, even WBRT or WBRT + SIB had well OS and iLC with tolerable side reactions. Its suggestion needed to be considered that brain radiotherapy is combined with ICIs. In terms of technology optimization, advanced radiotherapy techniques such as IMRT, image‐guided radiotherapy, and SRS are expected to deliver radiotherapy doses more precisely and reduce the risk of severe adverse events [25]. In the future, further evaluation of brain radiotherapy combined with ICIs in SCLC is required.

Our research has several limitations. First of all, all the studies included in this study are retrospective and are therefore susceptible to selection bias. As the difference in intracranial and extracranial disease burden between the WBRT and WBRT+SIB treatment cohorts suggests, patients with a more favorable survival prognosis and limited metastatic burden may be selected to receive WBRT+SIB treatment, which may exaggerate the total survival and other aggregated estimates of results In addition, most studies exclude patients who have received PCI in the past, which prevents us from extending our findings to patients receiving this standard treatment. Second, some of our analyses combined single‐arm result estimates, which provided accuracy for the prognosis of WBRT or WBRT+SIB combined ICIs, but the cross‐trial comparison should be interpreted with caution. Third, the sample size is relatively small, which leads to a certain degree of deviation. Large‐sample prospective studies should be further conducted to verify the conclusions.

## Author Contributions

J.X., Y.Y., and T.C., performed data acquisition, the statistical nalysis and drafted the manuscript, the three authors contributed equally to the study. D.L., and Y.Y., performed data acquisition and the statistical analysis. Y.Y., and L.X., critically reviewed the manuscript. All authors contributed to the article and approved the submitted version.

## Ethics Statement

The studies involving humans were approved by the ethics committee of Tianjin Medical University Cancer Hospital. The studies were conducted in accordance with local legislation and institutional requirements. Written informed consent for participation was not required from the participants or the participants' legal guardians/next of kin in accordance with the national legislation and institutional requirements.

## Conflicts of Interest

The authors declare no conflicts of interest.

## References

[1] M. Pizzato , M. Li , J. Vignat , et al., “The Epidemiological Landscape of Thyroid Cancer Worldwide: GLOBOCAN Estimates for Incidence and Mortality Rates in 2020,” Lancet Diabetes and Endocrinology 10, no. 4 (2022): 264–272, 10.1016/S2213-8587(22)00035-3. 35271818

[2] I. Gomez‐Randulfe , R. Leporati , B. Gupta , S. Liu , and R. Califano , “Recent Advances and Future Strategies in First‐Line Treatment of ES‐SCLC,” European Journal of Cancer 200 (2024): 113581, 10.1016/j.ejca.2024.113581. 38301317

[3] C. L. Chiang , H. C. Yang , Y. T. Liao , et al., “Treatment and Survival of Patients With Small Cell Lung Cancer and Brain Metastasis,” Journal of Neuro‐Oncology 165, no. 2 (2023): 343–351, 10.1007/s11060-023-04512-2. 37983003

[4] B. Mennecier , J. Khalifa , R. Descourt , L. Greillier , C. Naltet , and L. Falchero , “Real‐Life Clinical Management Patterns in Extensive‐Stage Small Cell Lung Cancer Across France: A Multi‐Method Study,” BMC Cancer 24, no. 1 (2024): 421, 10.1186/s12885-024-12117-9. 38580937 PMC10996204

[5] L. Paz‐Ares , M. C. Garassino , Y. Chen , et al., “Durvalumab ± Tremelimumab + Platinum‐Etoposide in Extensive‐Stage Small Cell Lung Cancer (CASPIAN): Outcomes by PD‐L1 Expression and Tissue Tumor Mutational Burden,” Clinical Cancer Research 30, no. 4 (2024): 824–835, 10.1158/1078-0432.CCR-23-1689. 37801329 PMC10870117

[6] W. Tian , X. Chu , G. Tanzhu , and R. Zhou , “Optimal Timing and Sequence of Combining Stereotactic Radiosurgery With Immune Checkpoint Inhibitors in Treating Brain Metastases: Clinical Evidence and Mechanistic Basis,” Journal of Translational Medicine 21, no. 1 (2023): 244, 10.1186/s12967-023-04089-4. 37020242 PMC10077682

[7] A. Louveau , I. Smirnov , T. J. Keyes , et al., “Structural and Functional Features of Central Nervous System Lymphatic Vessels,” Nature 523, no. 7560 (2015): 337–341, 10.1038/nature14432. 26030524 PMC4506234

[8] Y. Lan , M. Moustafa , M. Knoll , et al., “Simultaneous Targeting of TGF‐β/PD‐L1 Synergizes With Radiotherapy by Reprogramming the Tumor Microenvironment to Overcome Immune Evasion,” Cancer Cell 39, no. 10 (2021): 1388–1403.e10, 10.1016/j.ccell.2021.08.008. 34506739

[9] S. Rieken , “Stereotactic Radiosurgery for Patients With Small‐Cell Lung Cancer Brain Metastases,” Lancet Oncology 23, no. 7 (2022): 832–833, 10.1016/S1470-2045(22)00301-1. 35644162

[10] Q. Zeng , X. Chu , G. Xiao , et al., “The Optimal Radiotherapy Strategy for Patients With Small Cell Lung Cancer and Brain Metastasis: A Retrospective Analysis,” CNS Neuroscience & Therapeutics 30, no. 11 (2024): e70102, 10.1111/cns.70102. 39500635 PMC11537770

[11] K. Gaebe , A. Y. Li , A. Park , et al., “Stereotactic Radiosurgery Versus Whole Brain Radiotherapy in Patients With Intracranial Metastatic Disease and Small‐Cell Lung Cancer: A Systematic Review and Meta‐Analysis,” Lancet Oncology 23, no. 7 (2022): 931–939, 10.1016/S1470-2045(22)00271-6. 35644163

[12] R. Rittberg , S. Banerji , J. O. Kim , S. Rathod , and D. E. Dawe , “Treatment and Prevention of Brain Metastases in Small Cell Lung Cancer,” American Journal of Clinical Oncology 44, no. 12 (2021): 629–638, 10.1097/COC.0000000000000867. 34628433

[13] C. G. Rusthoven , M. Yamamoto , D. Bernhardt , et al., “Evaluation of First‐Line Radiosurgery vs Whole‐Brain Radiotherapy for Small Cell Lung Cancer Brain Metastases: The FIRE‐SCLC Cohort Study,” JAMA Oncology 6, no. 7 (2020): 1028–1037, 10.1001/jamaoncol.2020.1271. 32496550 PMC7273318

[14] D. W. Andrews , C. B. Scott , P. W. Sperduto , et al., “Whole Brain Radiation Therapy With or Without Stereotactic Radiosurgery Boost for Patients With One to Three Brain Metastases: Phase III Results of the RTOG 9508 Randomised Trial,” Lancet 363, no. 9422 (2004): 1665–1672, 10.1016/S0140-6736(04)16250-8. 15158627

[15] C. Bailleux , L. Eberst , and T. Bachelot , “Treatment Strategies for Breast Cancer Brain Metastases,” British Journal of Cancer 124, no. 1 (2021): 142–155, 10.1038/s41416-020-01175-y. 33250512 PMC7782834

[16] J. Zhong , A. D. Waldman , S. Kandula , et al., “Outcomes of Whole‐Brain Radiation With Simultaneous In‐Field Boost (SIB) for the Treatment of Brain Metastases,” Journal of Neuro‐Oncology 147, no. 1 (2020): 117–123, 10.1007/s11060-020-03405-y. 31970594

[17] B. S. H. Chia , J. Y. Leong , A. L. K. Ong , et al., “Randomised Prospective Phase II Trial in Multiple Brain Metastases Comparing Outcomes Between Hippocampal Avoidance Whole Brain Radiotherapy With or Without Simultaneous Integrated Boost: HA‐SIB‐WBRT Study Protocol,” BMC Cancer 20, no. 1 (2020): 1045, 10.1186/s12885-020-07565-y. 33126867 PMC7602352

[18] D. Qing , B. Zhao , Y. C. Zhou , H. L. Zhu , and D. Y. Ma , “Whole‐Brain Radiotherapy Plus Sequential or Simultaneous Integrated Boost for the Treatment of a Limited Number of Brain Metastases in Non‐Small Cell Lung Cancer: A Single‐Institution Study,” Cancer Medicine 9, no. 1 (2020): 238–246, 10.1002/cam4.2696. 31749325 PMC6943150

[19] J. W. Welsh , J. V. Heymach , C. Guo , et al., “Phase 1/2 Trial of Pembrolizumab and Concurrent Chemoradiation Therapy for Limited‐Stage SCLC,” Journal of Thoracic Oncology 15, no. 12 (2020): 1919–1927, 10.1016/j.jtho.2020.08.022. 32916308 PMC10600713

[20] S. Buriolla , G. Pelizzari , C. Corvaja , et al., “Immunotherapy in NSCLC Patients With Brain Metastases,” International Journal of Molecular Sciences 23, no. 13 (2022): 7068, 10.3390/ijms23137068. 35806080 PMC9267075

[21] Y. Yang , L. Deng , Y. Yang , et al., “Efficacy and Safety of Combined Brain Radiotherapy and Immunotherapy in Non‐Small‐Cell Lung Cancer With Brain Metastases: A Systematic Review and Meta‐Analysis,” Clinical Lung Cancer 23, no. 2 (2022): 95–107, 10.1016/j.cllc.2021.06.009. 34284948

[22] Y. Tian , J. Ma , X. Jing , et al., “Radiation Therapy for Extensive‐Stage Small‐Cell Lung Cancer in the Era of Immunotherapy,” Cancer Letters 541 (2022): 215719, 10.1016/j.canlet.2022.215719. 35597478

[23] L. Horn , A. S. Mansfield , A. Szczęsna , et al., “First‐Line Atezolizumab Plus Chemotherapy in Extensive‐Stage Small‐Cell Lung Cancer,” New England Journal of Medicine 379, no. 23 (2018): 2220–2229, 10.1056/NEJMoa1809064. 30280641

[24] C. M. Rudin , M. M. Awad , A. Navarro , et al., “Pembrolizumab or Placebo Plus Etoposide and Platinum as First‐Line Therapy for Extensive‐Stage Small‐Cell Lung Cancer: Randomized, Double‐Blind, Phase III KEYNOTE‐604 Study,” Journal of Clinical Oncology 38, no. 21 (2020): 2369–2379, 10.1200/JCO.20.00793. 32468956 PMC7474472

[25] A. Levy , A. Botticella , C. Le Péchoux , and C. Faivre‐Finn , “Thoracic Radiotherapy in Small Cell Lung Cancer‐a Narrative Review,” Transl Lung Cancer Res 10, no. 4 (2021): 2059–2070, 10.21037/tlcr-20-305. 34012814 PMC8107758

